# Supplementary material for: The anti-tumor effect of trifluridine via induction of aberrant mitosis is unaffected by mutations modulating p53 activity
Source: Cell Death Discov. 2024 Jul 2;10:307. doi: 10.1038/s41420-024-02083-3 (PMC11219725; doi:10.1038/s41420-024-02083-3)
Supplement: Supplementary file 1 — Supplementary Figure [file 41420_2024_2083_MOESM1_ESM.pdf]

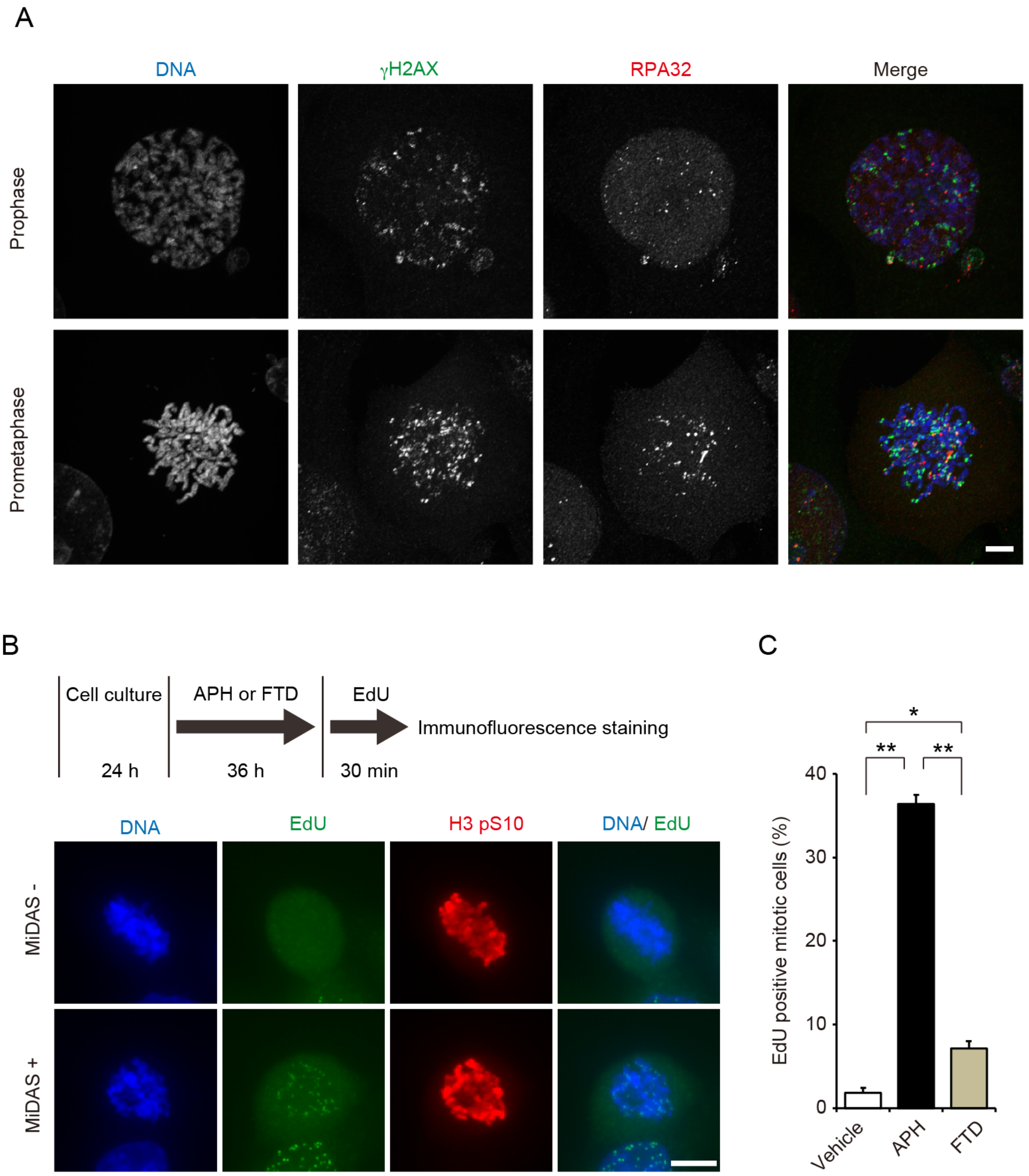

Supplementary Figure 1. The response of p53-KO cells to FTD treatment during mitosis.

A, Immunofluorescence images showing the localization of γH2AX/ RPA32 in FTD-treated mitotic cells. Cells were fixed and co-stained with the indicated antibodies. Scale bar, 10 μm. **B** and **C,** Representative images of mitotic DNA synthesis (MiDAS) and its experimental scheme are shown in B. Quantification of EdU positive cells is shown in C. APH, aphidicolin. Data are means± s.d. from three independent experiments (≥ 250 cells per experiment). * $P < 0.001$, ** $P < 0.0001$Scale bar, 10μm.

A

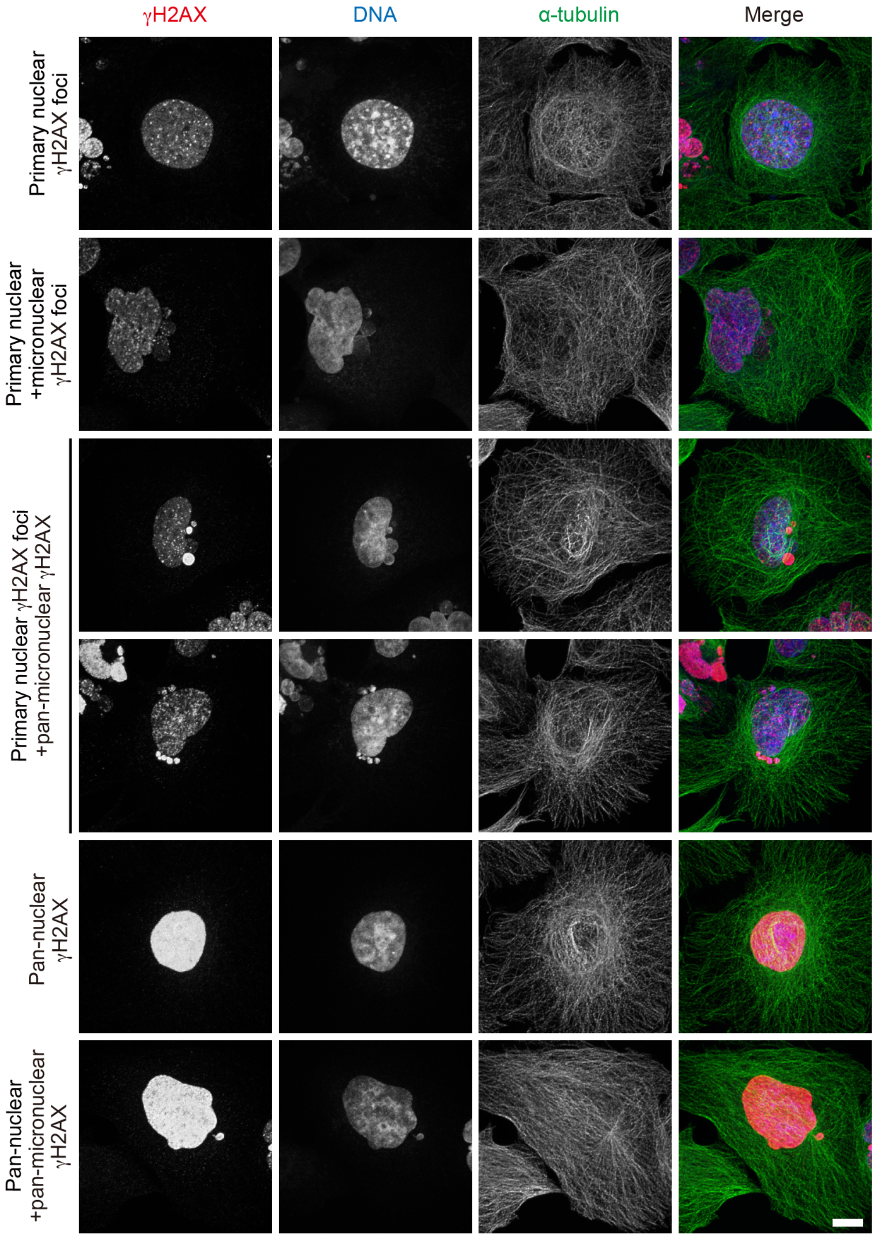

γH2AX
DNA
α-tubulin
Merge
Primary nuclear γH2AX foci
Primary nuclear +micronuclear γH2AX foci
Primary nuclear γH2AX foci +pan-micronuclear γH2AX
Pan-nuclear γH2AX
Pan-nuclear +pan-micronuclear γH2AX

B

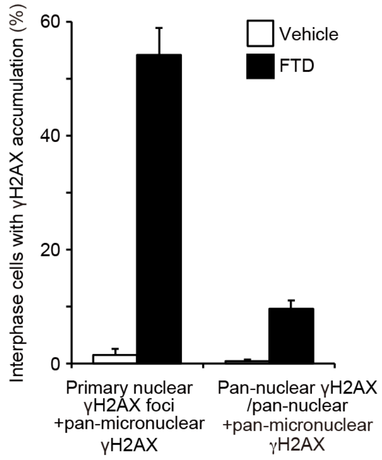

Interphase cells with γH2AX accumulation (%)
60
40
20
0
Vehicle
FTD
Primary nuclear γH2AX foci +pan-micronuclear γH2AX
Pan-nuclear γH2AX /pan-nuclear +pan-micronuclear γH2AX

Supplementary Figure 2. γH2AX accumulation in FTD-treated cells.
A, Immunofluorescence images showing the localization of γH2AX in FTD-treated cells. Cells were treated with FTD for 72 h, fixed and co-stained with the indicated antibodies. Scale bar, 10 μm. **B,** Quantification of proportion of cells exhibiting γH2AX accumulation in primary nucleus or micronuclei. Data are means± s.d. from three independent experiments (≥ 250 cells per experiment). Scale bar, 10μm.

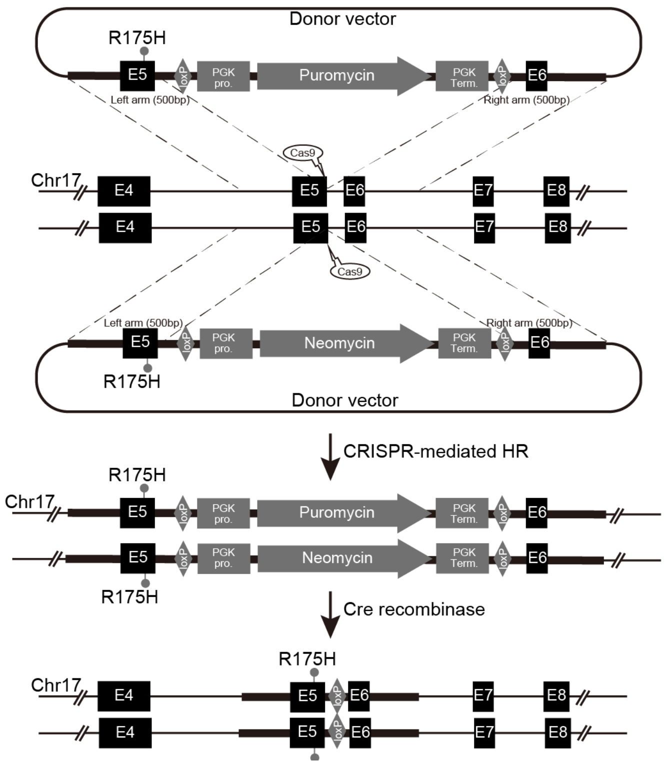

Supplementary Figure 3. Experimental scheme for generating p53-GOF mutation knock-in cells.

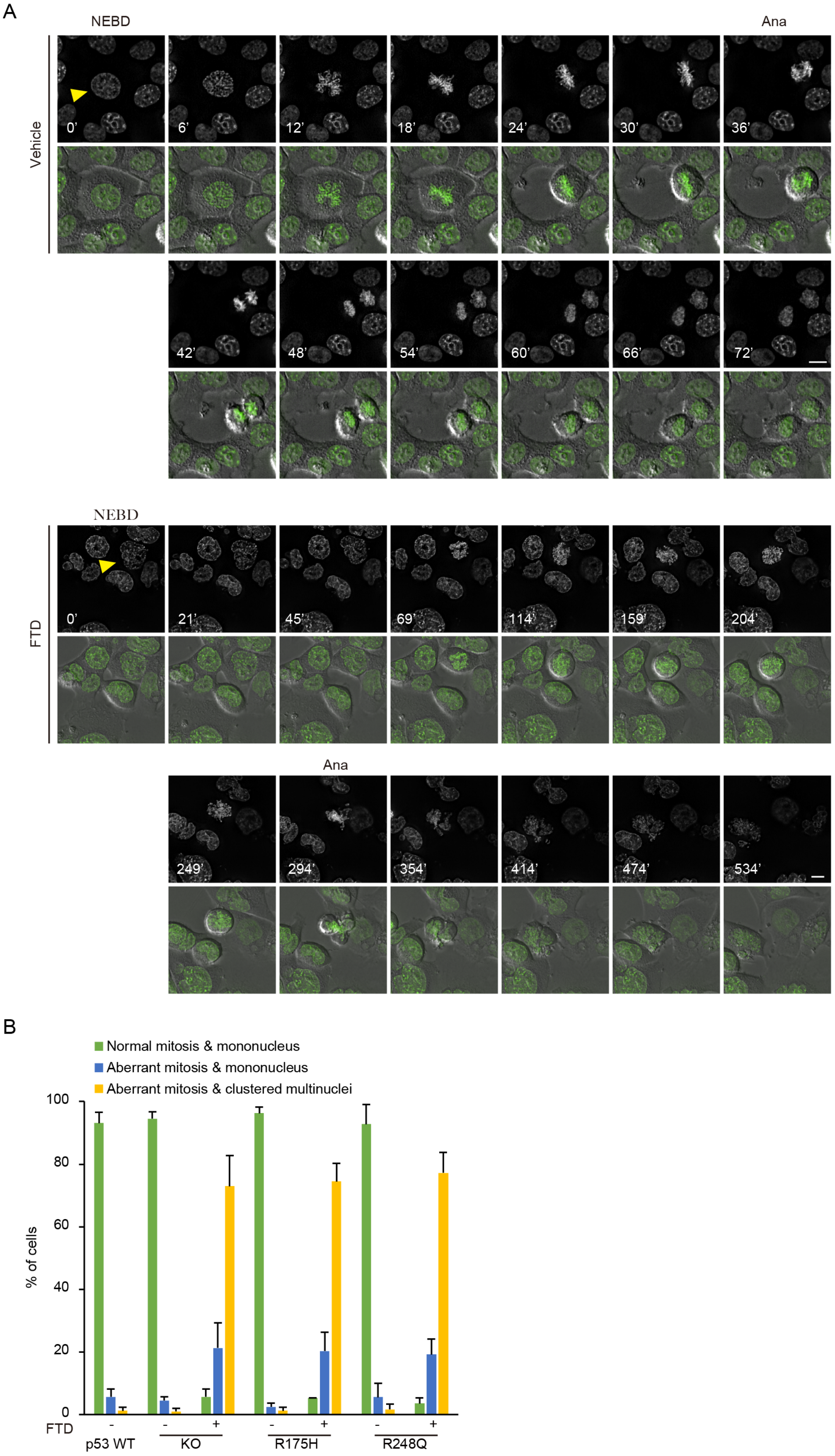
A
NEBD
Ana
Vehicle
0'
6'
12'
18'
24'
30'
36'
42'
48'
54'
60'
66'
72'
NEBD
FTD
0'
21'
45'
69'
114'
159'
204'
Ana
249'
294'
354'
414'
474'
534'
B
Normal mitosis & mononucleus
Aberrant mitosis & mononucleus
Aberrant mitosis & clustered multinuclei
% of cells
100
80
60
40
20
0
FTD
-
-
+
-
+
-
+
p53 WT
KO
R175H
R248Q

Supplementary Figure 4. Aberrant mitotic progression following FTD treatment in p53-GOF cells.
A, Selected frames from live-cell imaging of representative HCT116 p53-GOF cells expressing histone H2B-GFP treated with FTD. Time (minutes) after nuclear envelope breakdown (NEBD) is shown on the images. Arrowheads highlight representative cell images. Ana, onset of anaphase. Scale bar, 10 µm. **B,** Quantification of cells exhibiting aberrant mitotic progression and post-mitotic nuclear structure formation. Data are means± s.d. from three independent experiments (≥ 250 cells per experiment). Scale bar, 10µm.

Fig. 3B

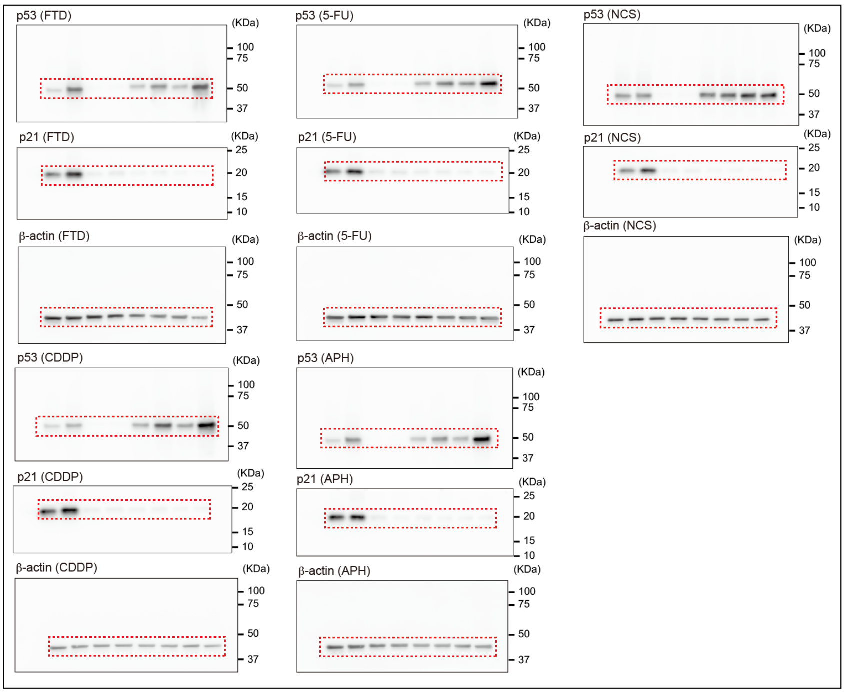

Fig. 3C

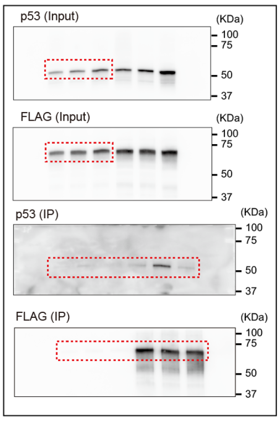

Fig. 4G

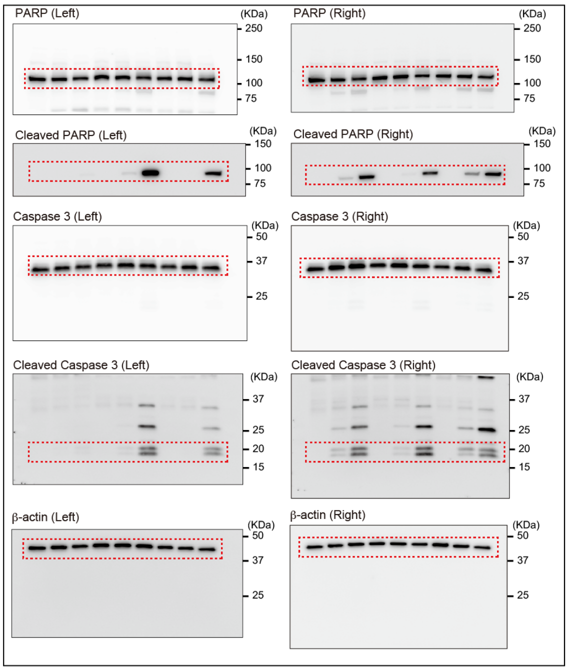

Fig. 4H

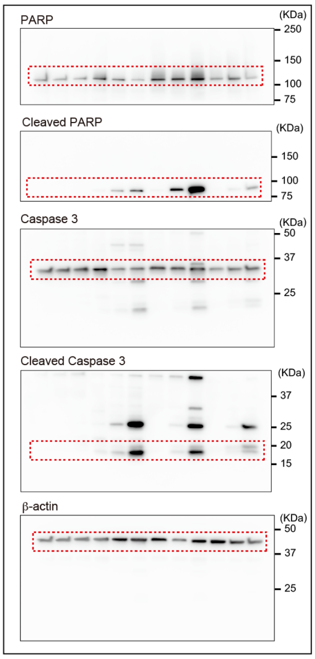

Supplementary Figure 5. Uncropped images of immunoblot.
Red boxes show cropped regions.
